# The Type II Secretory System Mediates Phage Infection in *Vibrio cholerae*

**DOI:** 10.3389/fcimb.2021.662344

**Published:** 2021-04-16

**Authors:** Huihui Sun, Ming Liu, Fenxia Fan, Zhe Li, Yufeng Fan, Jingyun Zhang, Yuanming Huang, Zhenpeng Li, Jie Li, Jialiang Xu, Biao Kan

**Affiliations:** ^1^ State Key Laboratory for Infectious Disease Prevention and Control, National Institute for Communicable Disease Control and Prevention, Chinese Center for Disease Control and Prevention, Beijing, China; ^2^ National Institute of Environment Health, Chinese Center for Disease Control and Prevention, Beijing, China; ^3^ School of Light Industry, Beijing Technology and Business University, Beijing, China

**Keywords:** bacteriophage, type II secretory system, receptor, EpsD, *Vibrio cholerae*

## Abstract

Attachment and specific binding to the receptor on the host cell surface is the first step in the process of bacteriophage infection. The lytic phage VP2 is used in phage subtyping of the *Vibrio cholerae* biotype El Tor of the O1 serogroup; however, its infection mechanism is poorly understood. In this study, we aimed to identify its receptor on *V. cholerae*. The outer membrane protein EpsD in the type II secretory system (T2SS) was found to be related to VP2-specific adsorption to *V. cholerae*, and the T2SS inner membrane protein EpsM had a role in successful VP2 infection, although it was not related to adsorption of VP2. The tail fiber protein gp20 of VP2 directly interacts with EpsD. Therefore, we found that in *V. cholerae*, in addition to the roles of the T2SS as the transport apparatus of cholera toxin secretion and filamentous phage release, the T2SS is also used as the receptor for phage infection and probably as the channel for phage DNA injection. Our study expands the understanding of the roles of the T2SS in bacteria.

## Introduction

Cholera is a severe intestinal infectious disease caused by toxigenic *Vibrio cholerae*, which still seriously attacks human health in undeveloped countries. Cholera outbreaks and endemics occur mainly due to the ingestion of contaminated water and food (Sack et al., 2004; Weil and Ryan, 2018). Successfully colonized *V. cholerae* in the host small intestine may express a number of virulence factors in response to host signals, including cholera toxin (Reidl and Klose, 2002; Yang et al., 2013; Liu et al., 2016), thus causing vomiting and watery diarrhea symptoms. *V. cholerae* can be divided into more than 200 serogroups according to the diversity of surface O antigens; currently, only toxigenic O1 and O139 serogroup strains are believed to cause cholera epidemics or pandemics (Faruque et al., 1998), and O1 strains can be further classified into classical biotype and El Tor biotype.

Bacteriophage or phage is a general term for viruses that can infect bacteria, causing lysis or lysogenic conversion of the infected bacterial host according to the characteristics of virulent or lytic phages and temperate phages (Hatfull and Hendrix, 2011; Salmond and Fineran, 2015). Interactions between phages and bacteria result in the modulation and biodiversity of bacterial communities in the environment and host. In *V. cholerae*, phages may cause the proliferation and decline of the different clones in the environment and may contribute to the disappearance of epidemics or the appearance of new epidemics caused by new clones (Faruque et al., 2005; Jensen et al., 2006). Lytic phages can also be used in the subtyping of bacteria and even to eliminate drug-resistant pathogens in the clinic (Kazmierczak et al., 2014).

The first step for phages to infect the host bacteria is attachment to the specific receptors of the host cells. Tailed phages use a broad range of receptor-binding proteins to specifically interact with their cognate bacterial cell surface receptors. These receptors are diverse in different host cells, and most components are localized on the bacterial cell surface, such as many porins of the outer membrane, lipopolysaccharide (LPS) (Rakhuba et al., 2010; Bertozzi Silva et al., 2016), and flagella or pili, which can also act as phage receptors (Harvey et al., 2018; McCutcheon et al., 2018). Identifying the receptors of phages may facilitate understanding of the infection and host range of the phages and resistance mutations of the bacteria since a sensitive host may evolve resistance to phages by receptor mutation, for example, under infection pressure from phages.

In a phage typing scheme for the El Tor biotype of O1 serogroup *V. cholerae*, five *V. cholerae* lytic phages (named VP1 to VP5 in turn) are used for the subtyping of O1 El Tor strains (Zhang et al., 2009). Lipopolysaccharide and outer membrane proteins have been found to be receptors of some typing phages (Zhang et al., 2009). In this study, we aimed to identify the receptor of the phage VP2. We adopted the transposon library strategy to identify the genes related to VP2 infection in *V. cholerae* and found a type II secretion system (T2SS), which has been identified for its importance as an extracellular protein transport apparatus for the secretion of cholera enterotoxin (CT) and release of the filamentous phage CTXΦ (Davis et al., 2000), also playing a vital role for VP2 adsorption and infection processes. Our study revealed a new role of T2SS, which is a common cross-membrane apparatus in many bacteria, and present a new insight for the function of this transporter.

## Materials and Methods

### Phage, Bacterial Strains, Plasmids, and Media

VP2 was isolated from the river water of Wenzhou, Zhejiang Province, in 1962. The bacterial strains and plasmids used in this study are listed in **Table 1**. *V. cholerae* O1 El Tor 16017 is the host of VP2 and is used for VP2 propagation. The *V. cholerae* El Tor strain N16961 is sensitive to the phage VP2 and was used as the wild-type strain in this study. For *in vitro* growth experiments, the bacteria were cultured in LB medium unless otherwise noted. Antibiotics were used at the following concentrations: ampicillin (Amp), 100 μg/mL; streptomycin (Sm), 100 μg/mL; kanamycin (Kan), 50 μg/mL; gentamicin (Gm), 20 μg/mL; tetracycline (Tc), 2 μg/mL and chloramphenicol (Cm), 30 μg/mL for *Escherichia coli* but 2 μg/mL for *V. cholerae*.

**Table 1:** Strains and plasmids used in this study.

Strains and plasmids	Relevant properties	Reference
***V. cholerae*** N16961	Spontaneous mutant of N16961, Inaba, Sm^r^	Laboratory collections
N*-*Δ*epsM*	*epsM* (VC2724) deletion of N16961, Sm^r^	This study
N*-*Δ*epsD*	*epsD* (VC2733) deletion of N16961, Sm^r^	This study
N*-*Δ*epsM-*C	N-Δ*epsM* strain complemented with pSRKTc-EpsM	This study
N*-*Δ*epsD-*C	N-Δ*epsD* strain complemented with pSRKGm-EpsD	This study
***E. coli***		
SM10*λpir*	*Kan, thi thr leu tonA lacY supE recA::RP4-2-TC::Muλpir*	
DH5α*λpir*	*sup E44, ΔlacU169 (ΦlacZΔM15), recA1, endA1, hsdR17, thi-1, gyrA96, relA1*	
XL1blue	*end*A1 *gyr*A96 *thi*-1 *hsd*R17 *sup*E44 *rel*A1 *lac*	
BTH101	*F-, cya-99, araD139, galE15, galK16, rpsL1 (Strr), hsdR2, mcrA1, mcrB1*	
**Plasmids**		
pSC123	Suicide plasmid carrying transposon; Kan^r^ Cm^r^	
pWM91	Suicide plasmid; *oriR oriT lacZ tetAR sacB*	
pWM91-Δ*epsM*	pWM91 carrying upstream and downstream fragments flanking *epsM*	This study
pWM91-Δ*epsD*	pWM91 carrying upstream and downstream fragments flanking *epsD*	This study
pUT18C (abbreviated as pT18C)	pUT18C-derived vector, designed to create C-terminal heterologous protein fusion, Amp^r^	
pKT25 (abbreviated as pT25)	*lac* promoter and the T25 fragment for C-terminal heterologous protein fusion. Kan^r^	
pSRKTc	*lac* promoter and *lacI^q^*, Tet^r^	
pSRKTc-EpsM	pSRKTc-derived, VC2724 (*epsM*), Tet^r^	This study
pSRKGm-EpsD	pSRKGm-derived, VC2733 (*epsD*), Tet^r^	This study
pT25-EpsD	pKT25-derived, *epsD*, Kan^r^	This study
pT18C-gp20	pUT18C-derived, *gp20*, Amp^r^	This study
pGEX-4T-1-EpsD	pGEX-4T-1-derived, *epsD*, Amp^r^	This study
pET30a-gp20	pET30a(+)-derived, *gp20*, Kan^r^	This study

### Screening of VP2-Resistant Mutants

*E. coli* SM10*λpir* (Chiang and Mekalanos, 2000) bearing the plasmid pSC123 (Simon et al., 1983) named SM10-123, was used as the donor strain, and conjugation was performed by using the VP2-sensitive *V. cholerae* strain N16961 as the recipient to obtain a transposon insertion library. The transposon mutation pool was mixed with a VP2 suspension (10^8^ PFU/mL) at a ratio of 1:5 and incubated for 10 min at 37°C. The resulting cultures were spread onto LB agar plates with Kan and Sm. The resistant strains were subsequently verified by double-layer plaque assay as described previously (Frost et al., 1999), and the confirmed nonlysed strains were selected as candidates for verifying transposon insertion location.

Arbitrary PCR (Judson and Mekalanos, 2000) was performed with two rounds to identify the transposon insertion site on the chromosome. In the first round, the chromosomal DNA of candidates was used as amplification templates, and the primers ARB-1, ARB-6, and 123-3 (**Table 2**) were used in amplification. PCR was performed under the following conditions: 95°C for 5 min; six cycles of 94°C for 30 s, 30°C for 30 s, and 72°C for 1 min; 30 cycles of 94°C for 30 s, 55°C for 30 s, and 72°C for 1 min; and 72°C for 5 min. The second round of PCR amplified the PCR product of the first round with the primers 123-4 and ARB-2 (**Table 2**). Amplification conditions were as follows: 30 cycles of 94°C for 30 s, 55°C for 30 s, and 72°C for 1 min, followed by 72°C for 5 min. The amplicons were sequenced using the primer 123-4. The transposon insertion sites were confirmed by comparing the sequencing results with the N16961 reference sequence.

**Table 2:** Primers used in this study.

Primer	Sequence (5’–3’)^a^	Destination
VC2724-UP-XhoI-5’	CCGCTCGAGAGTGGTACCGGTAGCGGTAG	pWM91-Δ*eps*M
VC2724-UP-3’	CAAGTAAGGAGAACAGCCGCGCGGCGAAATGAT	
VC2724-DOWN-5’	CGCCGCGCGGCTGTTCTCCTTACTTGGGCTTCA	
VC2724-DOWN-NotI-3’	AAGGAAAAAAGCGGCCGCCCAAGAGTGGCGTTATGAGC	
VC2733-UP-XhoI-5’	CCGCTCGAGTGGCCATCACTTTGACCCGT	
VC2733-UP-3’	GTTCCCAGTGAAACAACCAGCTCGCAAGTGGAA	
VC2733-DOWN-5’	TGCGAGCTGGTTGTTTCACTGGGAACTCCCTTG	pWM91-Δ*epsD*
VC2733-DOWN-NotI-3’	AAGGAAAAAAGCGGCCGCCGACTTGGAGCCCCACGTTA	
123-3	TCACCAACTGGTCCACCTAC	arbitrary PCR
123-4	CGCTCTTGAAGGGAACTATG	
ARB1	GGCCACGCGTCGACTAGTACNNNNNNNNNNGATAT	
ARB6	GGCCACGCGTCGACTAGTACNNNNNNNNNNACGCC	
ARB2	GGCCACGCGTCGACTAGTAC	
EpsM- NdeI -F	GGAATTCCATATGATGAAAGAATTATTGGCTCCTGTG	pSRKTc-EpsM
EpsM- XbaI -R	GCTCTAGATCAGCCTCCACGCTTCAGTTG	
EpsD-XbaI-F	GCTCTAGACATTGCTTGGCTTCCATCTG	
EpsD-NdeI-R	GGAATTCCATATGAGGGAGTTCCCAGTGAAATA	EpsD
gp20-BamHI-F	CGCGGATCCCATGACTATCCAGAACAAAGAACC	pT18-gp20
gp20-KpnI-R	CGGGGTACCCGAACGTCCGTAAAATACAAGTT	
nT25-EpsD-BamHI-F	CGCGGATCCCAACGAGTTTAGCGCCAGCTTT	pT25-EpsD
nT25-EpsD-KpnI-R	CGGGGTACCCGTTGCTTGGCTTCCATCTGCTCAA	
EpsD-BamHI-F	CGCGGATCCCGCCGTGCGCAAGTGTTGA	pGEX-4T-1-EpsD
EpsD-XhoI-R	CCGCTCGAGTCATTGCTTGGCTTCCATCTGC	
gp20-BamHI-F	CGCGGATCCATGACTATCCAGAACAAAGAA	pET-30a-gp20
gp20-HindIIII-R	CCCAAGCTTTTAAACGTCCGTAAAATACAAG	

^a^Restriction sites are underlined.

### Construction of Gene Deletions and Complementation

In-frame deletions were constructed by cloning the regions flanking target genes into the suicide vector pWM91 containing a *sacB* counterselectable marker (Metcalf et al., 1996). The recombinant plasmid was transformed into the strain SM10*λpir* and introduced into *V. cholerae* N16961 by conjugation. Double-crossover recombination mutants were selected using 10% sucrose plates at 22°C and confirmed *via* PCR and sequencing. The plasmids overexpressing *epsM* and *epsD* were obtained by cloning the complete *epsM* into a pSRKTc plasmid and *epsD* genes into a pSRKGm plasmid (Khan et al., 2008).

The primers used for the construction are listed in **Table 2**.

### VP2 Lysis Assay and Efficiency-of-Plating Assay

A double-agar overlay plaque assay (Korotkov and Sandkvist, 2019) was used to examine the lysis status of VP2 on *V. cholerae*. Briefly, 100 μL of exponential cultures of wild-type and *epsM* and *epsD* deletion mutant strains and their complements were mixed with 0.6% soft agar medium and poured on top of bottom agar. Ten microliters from a phage suspension containing approximately 10^8^ PFU was spotted in the middle of the lawn of bacteria, incubated overnight at 37°C and imaged. Each strain experiment was repeated three times. Bacterial strains were considered sensitive to the phage if the degree of lysis was observed as complete clearing. In contrast, bacterial strains were considered resistant if there was no effect of the phage on bacterial growth. When indicated, 500 μM IPTG was included in the medium to induce gene expression.

The efficiency-of-plating (EOP) assay was also conducted to quantitate the lysis ability of VP2 as previously described with some modifications (Yang et al., 2020). In brief, the wild-type strain, *epsM* and *epsD* deletion mutant strains and their complements (10^8^ CFU/mL) were mixed with 10^6^ PFU/mL concentrated VP2, incubated for 15 min, 10-fold dilutions of the mixtures were prepared with LB. For plating, 100 μl of diluents of the mixtures was add to 4 ml of 0.6% top agar and poured on the bottom agar, and the plate was incubated at 37°C overnight. The EOP value was calculated as the ratio of the number of lysis plaques produced on the bacterial lawn of the target to the number of plaques produced on the lawn of the host strain.

### Phage Adsorption Assays

A titer of at least 10^6^ PFU/mL concentrated VP2 was mixed separately with different *V. cholerae* variants (10^8^ CFU/mL), incubated for 3 min, 5 min and 10 min at 37°C, and then centrifuged at 6,000 rpm for 8 min. The residual phage titers of the supernatant were counted in 10^-3^ dilutions and tested by double-layer plaque assay. Phage without N16961 treatment was used as a control.

### Cloning, Expression and Purification of the VP2 Tail Filament Protein and T2SS EpsD

For a bacterial two-hybrid assay, the recombinant plasmids pKT25-*epsD* and pUT18C-gp20 were constructed as previously described (Fan et al., 2018). Briefly, two putative interacting proteins (EpsD and gp20) were genetically fused to two complementary fragments, T25 and T18, of the active domain of adenylate cyclase (CyaA) from *Bordetella pertussis* (Ladant, 1988). First, the *epsD* truncated sequence (lacking the signal peptide, encoding 25-674 aa) and gp20 complete sequence were amplified using the N16961 and VP2 genomes as templates, respectively. The PCR products of *epsD*_25-674_ and gp20 were digested with the *Bam*HI and *Kpn*I restriction enzymes and ligated into the corresponding pKT25 and pUT18C vectors digested with the same enzymes. The resulting plasmids were cotransformed into the *E. coli* Δ*cya* mutant BTH101 for subsequent study. All primers used are included in **Table 2**.

For pull-down assays, the truncated EpsD and complete gp20 genes were subcloned into prokaryotic expression vectors to obtain the corresponding proteins. A gene fragment of *epsD* (encoding 340-675 aa) was cloned into the pGEX-4T-1 vector and introduced into the overexpression strain *E. coli* BL21 (DE3), and protein purification was performed as described previously (Howard et al., 2019). Full-length gp20 was inserted into the pET-30a vector and induced in *E. coli* strain BL21 (DE3). Cells containing the pET-30a-gp20 recombinant plasmid were grown in LB medium until the OD_600_ reached 0.5-0.6, and 0.5 mM IPTG was added to induce expression overnight at 16°C. Cells were harvested, and the pellets were resuspended in buffer A (20 mM Tris-HCl, pH 9.0, 300 mM NaCl, supplemented with protease inhibitors). The cells were lysed by sonication and centrifuged at 12,000 rpm for 30 min at 4°C. The His6-tagged protein gp20 was purified using Ni^2+^ resin (Invitrogen), and the elution samples were dialyzed in buffer B (20 mM Tris-HCl, pH 9.0, 300 mM NaCl) overnight and were used in interaction assays with GST-tagged EpsD. The protein concentration was determined using Pierce’s BCA Protein Assay Reagent Kit. All primers and restriction enzymes used are listed in **Table 2**.

### Binding Capacity of vp2 Tail Filament Protein With Wild-Type N16961 and N-*ΔepsD*

We fused gp20 to a His6 tag, mixed it with an Alexa Fluor 488-conjugated anti-His-tag monoclonal antibody, incubated it in the dark at room temperature and resuspended it in filter-sterilized phosphate-buffered saline (PBS). After the unbound antibodies were washed out fully with PBS, they were mixed with wild-type strains and the mutant strain N-Δ*epsD*. Antibodies and no antibodies were added to these two strains as controls. Samples were then analyzed using BD flow cytometry.

### Bacterial Two-Hybrid System for Analysis of the EpsD and gp20 Interaction

The *cyaA* mutant *E. coli* BTH101 strain containing pKT25-*epsD* and pUT18C-gp20 was cultured overnight and then subcultured to log phase in LB medium with shaking at 220 rpm at 37°C. The β-galactosidase activities were measured in the presence of different concentrations of isopropyl-beta-D-thiogalactopyranoside (IPTG) as the inducer as previously described (Yang et al., 2013). The leucine zipper of GCN4 (Karimova et al., 1998) was used as a positive control.

### Ni^2+^-Affinity Pull-Down of gp20 and EpsD

Mixtures of 0.3 mg of His6-tagged gp20 protein and 0.1 mg of GST-tagged EpsD protein were rotated at room temperature for 2 h, bound to Ni^2+^ resin and incubated for 1 h at 4°C. Mixtures of His6-tagged gp20 protein and GST protein were used as negative controls.

The Ni^2+^ resin was washed extensively to remove nonspecifically bound proteins, and then the bound proteins were eluted with elution buffer containing 300 mM imidazole, 10 mM Tris-HCl and 500 mM NaCl (pH 8.0). Five micrograms of each sample were submitted to 12% SDS-PAGE and Western blot analysis. Unless otherwise noted, all samples were boiled for 10 min in SDS loading buffer before separation. After electrophoresis, proteins on the gels were transferred to PVDF membranes (Immobilon-P, Millipore). Both mouse anti-GST and anti-His6 tag monoclonal antibodies (Tiangen Biotech, Beijing) were used in the Western blot analysis. An anti-mouse peroxidase-conjugated AffiniPure IgG (H+L) secondary antibody (Zhong Shan Jin Qiao, Beijing) was used for protein detection.

## Results and Discussion

### Components of the Type II Secretion System Were Involved in VP2 Infection

**Figure 1:**
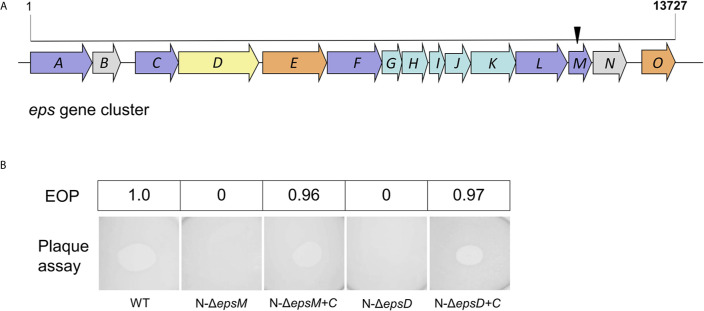
Transposon insertion and VP2 infection of the mutants. **(A)** Transposon insertion site of VP2-resistant mutants in the gene cluster of the T2SS in N16961. The *eps* genes are colored according to the classification of functions and positions of the proteins in T2SS in *V. cholerae* (Korotkov et al., 2012), including inner-membrane plateform proteins (light blue), outer-membrane secretin (yellow), pseudopilin (light green), others (light brown) and unknown (grey). The black arrow represents the site where the transposon was inserted into the *epsM*. **(B)** Detection of VP2 infection in *V. cholerae* mutants by double-layer plaque assay and EOP assay. The wild-type *V. cholerae* El Tor strain N16961 was used as the control for plaque formation. N*-*Δ*epsM* showed VP2 resistance (no plaque formation). The strain Δ*epsM*-C, carrying the EpsM expression plasmid cloned into the strain N-Δ*epsM*, was sensitive to VP2. N*-*Δ*epsD* showed VP2 resistance (no plaque formation). The strain Δ*epsD*-C, carrying the EpsD expression plasmid cloned into the strain N-Δ*epsD*, was sensitive to VP2. The values of EOP were shown in the top half of the figure.

To identify *V. cholerae* genes related to the resistance to VP2 infection phenotype, a selection strategy of phage-resistant mutants generated by transposon insertion was used. The pool of transposon mutants from the VP2-sensitive strain N16961 was generated by conjugation with SM10-123 carrying the plasmid pSC123. Then, the phage VP2 was added into this pool to lyse the mutant cells sensitive to VP2 infection, whereas the mutants resistant to VP2 survived. Possible resistant colonies were selected on agar and further confirmed for resistance to VP2 infection with double-layer agar. Eighteen candidates resistant to VP2 were collected. Each candidate was amplified through arbitrary PCR, and the amplicon was sequenced to verify the transposon location on the chromosome of the resistant strains. A total of six different insertion sites were identified, including in the coding sequences of the genes VCA0904 (H+/gluconate symporter and related permease), VC1936 (phosphatidate cytidylyltransferase), VC0718 (DNA recombination-dependent growth factor C), VC0420 (conserved hypothetical protein CHP02099), VCA0863 (lipase, Pla-1/cef, extracellular), and VC2724 (EpsM). Among these genes, VC2724 is located in the gene cluster of the T2SS, correspondingly coding for the inner membrane protein EpsM, which is located in the membrane (**Figure 1A**). For the phage receptor identification purpose of this study, we first selected the membrane-located protein EpsM as a candidate.

We further constructed the mutant strain N-Δ*epsM* with in-frame deletion of *epsM* from the wild-type strain N16961 and the complement strain N*-*Δ*epsM-*C carrying the intact *epsM* gene in the pSRKTc plasmid (**Table 2**). Phage lysis assays with both strains showed that N-Δ*epsM* was not lysed by VP2 (**Figure 1B**), whereas the strain N*-*Δ*epsM-*C carrying complementary *epsM* restored the sensitivity to VP2 (**Figure 1B**). EOP assay was consistent with the phage lysis assays (**Figure 1B**). Both assays showed that the *V. cholerae* T2SS protein EpsM is needed for VP2 infection.

### The Outer Membrane-Localized Protein EpsD of T2SS Could Adsorb VP2

T2SS is a cell envelope-spanning macromolecular complex that is prevalent in gram-negative bacterial species and serves as the predominant virulence mechanism of many bacteria (Johnson et al., 2006). The system is composed of a core set of highly conserved proteins that assemble an inner membrane platform, a periplasmic pseudopilus and an outer membrane complex termed the secretin (Johnson et al., 2006). The model of the T2SS machine includes an outer membrane protein, the secretin EpsD, the first subdomain of which is related to domains in phage tail filament proteins and outer membrane TonB-dependent receptors (Johnson et al., 2006). We suspected that EpsD might be involved in the interaction of *V. cholerae* with the phage VP2. Subsequently, we constructed the *epsD* deletion strain N-Δ*epsD* and the complementary strain N-Δ*epsD-*C (containing intact *epsD* in the plasmid pSRK-Gm) to determine their sensitivities to phage VP2. VP2 infection assays showed that the *epsD* mutant strain could not be lysed by VP2, while the strain N-Δ*epsD-*C could be lysed (**Figure 1B**), showing the important role of EpsD in VP2 infection; therefore, it could be expected that EpsD possibly acts as the receptor for VP2.

**Figure 2:**
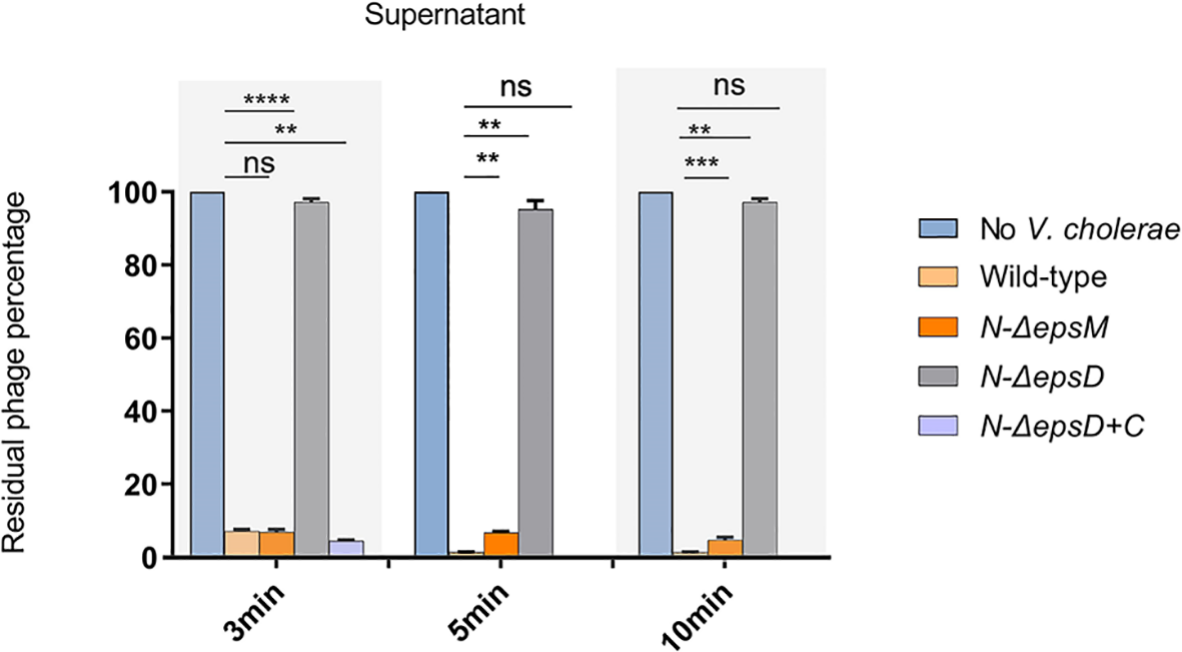
VP2 adsorption by the wild-type strain N16961 and its mutants. The VP2 phage (10^6^ CFU/mL) was mixed with fresh N16961 culture, N-*ΔepsM*, N-*ΔepsD*, N-*ΔepsD+*C (10^8^ CFU/mL) respectively for 3 min, 5min,10 min at 37°C, and then, each sample was centrifuged at 6000 rpm for 8 min. LB culture medium containing only VP2 phage was used as a negative control, and the phage titer in the control supernatant was set to 100%. The experiment was repeated three times. The mean of three independent assays was shown and error bars represent the standard deviation. **P =0.0026, ***P = 0.0001, ****P <0.0001, ns, no significance (Student’s *t* test).

The role of EpsD in VP2 infection was also determined by phage adsorption assays to detect the binding ability of the phage VP2 to the *epsD* and *epsM* mutant strains and their corresponding complementary strains. VP2 particles were mixed with the wild-type strain N16961 for 3 min, and after centrifugation, the remaining supernatant had a much lower phage titer than the control, which was determined by a PFU assay (**Figure 2**), showing that the sensitive strain N16961 had strong adsorption of VP2. When VP2 was mixed with the strain N-Δ*epsD*, its obvious VP2 titer difference in the supernatant was found when compared to the sensitive strain N16961 (**Figure 2**), indicating that VP2 did not bind to the *epsD* mutant. Such adsorption could be restored by complementing the plasmid carrying intact *epsD* into N-Δ*epsD* (strain N-Δ*epsD-*C, **Figure 2**). The VP2 adsorption efficiency of the *epsM* deletion strain N-Δ*epsM* was similar to that of the wild-type strain N16961. These data suggested that EpsD could adsorb VP2 but EpsM could not, which could be explained by their different membrane locations in the T2SS apparatus in *V. cholerae*.

### VP2 Adsorbed to EpsD of *V. cholerae* Through Its Tail Fiber Protein gp20

**Figure 3:**
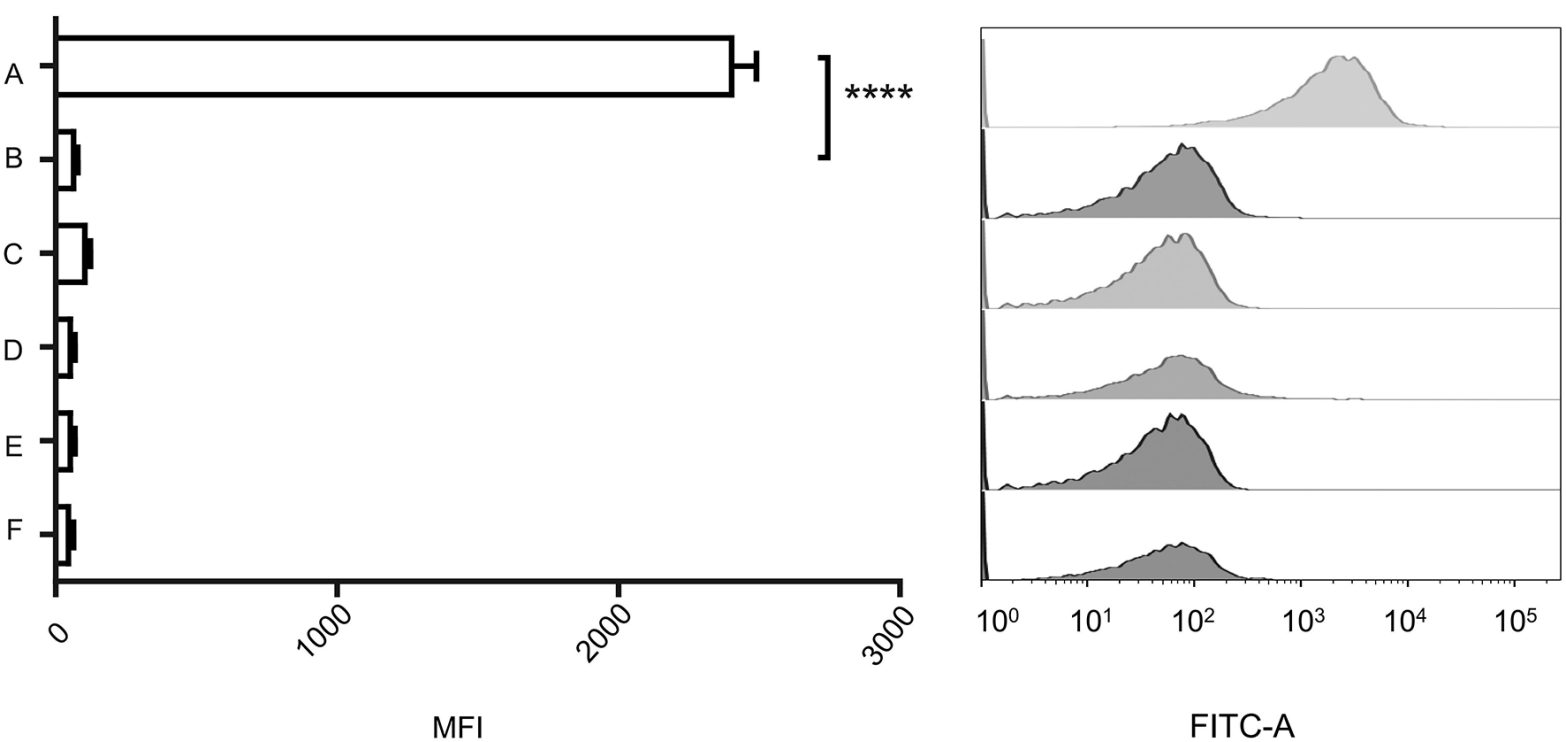
Binding capacity of the vp2 tail filament protein with wild-type N16961 and N-*ΔepsD*. (A): Wild type+ His6-tagged gp20+anti-His6 tag antibody; (B): N-*ΔepsD* + His6-tagged gp20+anti-His6 tag antibody; (C): wild type +anti-His6 tag antibody; (D): N-*ΔepsD* +anti-His6 tag antibody; (E): wild type; (F): N-*ΔepsD*. Ten micrograms of His6-tagged gp20 protein and 10 μg of Alexa Fluor 488-conjugated anti-His-tag monoclonal antibody (product # MA1-21315-A488) were mixed and incubated for 30 min in the dark at room temperature and then transferred to the wild-type N16961 and N-*ΔepsD* strains, with OD600 = 1. After induction at 37°C, induced cultures were washed twice with 1 mL of filter-sterilized PBS. Antibodies and no antibodies were added to these two strains as controls. Samples were analyzed using BD flow cytometry. The data shown on the left represent the geometric MFI. The data shown on the right represent the fluorescence intensity distribution of the bacteria analyzed in the experiment shown on the left. The mean of three independent assays is shown and error bars represent the standard deviation. ****P <0.0001 (Student’s *t* test).

The genome of VP2 has been sequenced previously in our laboratory (GenBank accession number: NC_005879). Here, we submitted the VP2 genome sequence to RAST (http://rast.nmpdr.org/rast.cgi) for the prediction of open reading frames (ORFs). The ORF *gp20* was predicted as the phage tail fiber protein gene, and then we expressed this protein as the ligand to detect its interaction with *V. cholerae*. To observe the binding ability of gp20 to the wild-type strain N16961 and N-Δ*epsD*, the expressed His6-tagged gp20 was labeled with an anti-His6 fluorescent antibody, mixed with the wild-type strain N16961 and mutant strain N-Δ*epsD*, and then analyzed using flow cytometry to measure the geometric mean fluorescence intensity (MFI). The MFI in the tests of the His6-tagged gp20/N16961 mixture was much higher than that of the His6-tagged gp20/N-Δ*epsD*, and the MFI of the latter was similar to that of the controls, representing the background fluorescence intensity, as shown in **Figure 3**, which indicated that no gp20 adsorbed to the *epsD* deletion mutant.

### The VP2 Tail Protein gp20 Interacted Directly With EpsD

**Figure 4:**
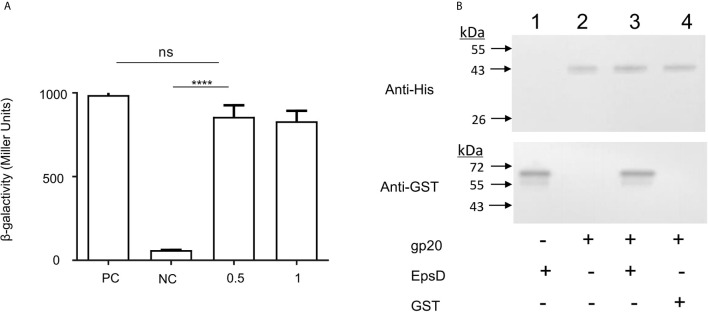
Binding capacity of the VP2 tail filament protein with wild-type N16961 and N-ΔepsD, and analysis of the interactions of EpsD with gp20. **(A)** Analysis the gp20-EpsD interaction by BACTH. PC, positive control (leucine zipper of GCN4); NC, negative control (vector plasmid only). The resulting recombinant plasmid pair pT25-gp20/pT18C-EpsD was co-transformed into BTH101 cells, and the β-galactosidase activity was measured. ****P <0.0001. ns, no significance. (Student’s *t* test). **(B)** Analysis of the interactions of EpsD with gp20 *in vitro*. Western blot analysis of His pull-down experiments was performed with GST-gp20 immobilized on Ni^2+^ resin, and the gp20 protein was incubated with GST for pull-down analysis as a negative control 0.3 mg of His6-tagged gp20 protein and 0.1 mg of GST-tagged EpsD protein were mixed, then the mixtures were rotated at room temperature for 2 h, bound to Ni2+ resin and incubated for 1 h at 4°C. Lane 1: His-gp20; 2: GST-EpsD; 3: Pull-down of His-gp20 and GST-EpsD; 4 Pull-down of His-gp20 + GST.

To determine the possible interaction between *V. cholerae* EpsD and gp20 of VP2, we performed mutual interaction assays between these two proteins by using a bacterial two-hybrid (BACTH) approach (Karimova et al., 1998). In this study, the recombinant plasmids pKT25-*epsD* and pUT18C-gp20 were constructed and co-transformed into the *E. coli* Δ*cya* mutant BTH101. The results showed that 0.5 mM IPTG could induce the maximum β-galactosidase activity, similar to that of 1.0 mM IPTG induction and the positive control (**Figure 4A**), suggesting that EpsD could interact with gp20.

In addition, pull-down assays were carried out to detect the interaction between gp20 and EpsD. His-tagged gp20 was immobilized on Ni2+ resin to capture GST-tagged EpsD. In the elution buffer, GST-tagged EpsD could be detected by Western blot (**Figure 4B**), further showing that gp20 can bind directly to EpsD.

Overall, we demonstrated that in *V. cholerae*, the outer membrane protein EpsD of T2SS acts as the receptor in phage VP2 infection. As an important secretion apparatus, T2SSs are widespread among gram-negative bacteria and contribute to pathogenesis in the host and environmental survival (Cianciotto and White, 2017; Korotkov and Sandkvist, 2019). The T2SS transports chitinase, lipase, hemagglutinin/protease and other proteases in some bacteria (Sandkvist, 2001; Evans et al., 2008; Sikora et al., 2011). *V. cholerae* may secrete cholera toxin (Sikora et al., 2011) and even mediate the release of the filamentous phage CTXФ through EpsD (Davis et al., 2000). In *E. coli*, the membrane protein PulD of the T2SS contributes to phage extrusion across the membrane and assembly of phages (Genin and Boucher, 1994; Russel, 1998; Sandkvist, 2001). In our study, in addition to these roles of T2SS, we showed that it may mediate lytic phage infection by the specific adsorption of VP2 to the outer membrane component EpsD. EpsM is a part of the interface between the regulating part and the rest Eps proteins of the T2SS (Abendroth et al., 2004). Combined with the dot assays and the phage adsorption assays, the inner membrane protein EpsM of T2SS plays a role in successful VP2 infection but does not affect VP2 binding to the host, suggesting that the intact T2SS structure probably acts as the phage DNA injection channel for crossing the outer and inner membranes of *V. cholerae*. The function of T2SS EpsD as a phage receptor has not been reported previously. Therefore, based on our study, the role of the T2SS in *V. cholerae* is expanded to the infection channel of the lytic phage, in addition to the transport of free proteins and release of filamentous phages.

## Data Availability Statement

The original contributions presented in the study are included in the article/supplementary material. Further inquiries can be directed to the corresponding author.

## Author Contributions

BK, HS and ML designed the study and wrote the paper. FF, ZheL and YF purified protein and performed the experiments. JZ, ZheL, and JX provided technical assistance and contributed to the preparation of the figures. JL cryopreserved and saved the strains. YH contributed to diagram modification and protein purification in the process of article modification. All authors contributed to the article and approved the submitted version.

## Funding 

This work was supported by the National Key Basic Research Program (2015CB554201) from Ministry of Science and Technology of the People’s Republic of China.

## Conflict of Interest

The authors declare that the research was conducted in the absence of any commercial or financial relationships that could be construed as a potential conflict of interest.
